# Boosting Human Papillomavirus Vaccination Rates: Protocol for a Randomized Controlled Trial of Awareness Interventions in Réunion Island

**DOI:** 10.2196/73366

**Published:** 2025-10-27

**Authors:** Julie Duclaud, Antoine Bertolotti, Emmanuel Chirpaz, Jessica Sambourg, Kevin Diallo, Vincent Balaya, Phuong Lien Tran

**Affiliations:** 1 Faculty of medicine University of Reunion Island Saint-Denis Réunion; 2 Department of gynecology and obstetrics Centre Hospitalier Universitaire de La Réunion Saint Pierre France

**Keywords:** human papillomavirus, HPV vaccine, primary prevention, middle school, serious game, peer learning

## Abstract

**Background:**

Human papillomavirus (HPV) is the most common sexually transmitted infection worldwide and imposes a significant public health burden. In 2019, HPV was responsible for approximately 620,000 cancer cases in women, 70,000 in men, and more than 300,000 deaths globally. Despite the proven efficacy of the vaccine, vaccination rates remain alarmingly low in certain regions of France. In Réunion Island, only 16% of girls and 9% of boys under 16 years old were fully vaccinated in 2024. This underscores the need for increased awareness, education, and outreach programs. Peer learning is well-established in health education, whereas serious game–style card games are newer and require further research. Both methods have been shown to improve knowledge on specific topics.

**Objective:**

The main objective of this study is to assess the impact of 2 awareness-raising strategies on increasing HPV vaccination rates among middle school students by actively involving them in the process.

**Methods:**

This protocol describes the design of a randomized, open-label, controlled trial aimed at evaluating the effectiveness of 2 awareness-raising interventions—peer learning and a card-based serious game—in improving HPV vaccination rates among middle school students in Réunion Island. The study will span an entire school year, beginning in August. Approximately 3600 students from 24 middle schools in Réunion Island will be included, with schools randomized into 3 groups of 8 each: (1) a control group receiving the existing national vaccination campaign initiated by the French public health institute; (2) ambassador classes, whose students will receive education about the HPV vaccine and later educate other students in the same school; and (3) serious game card group, where students can play and learn about HPV during a science class. The primary outcome will be the proportion of teenagers who initiate the vaccination process, compared across the 3 groups using appropriate statistical methods. Anonymized data will be collected at the end of the school year using social security records. Teenagers’ knowledge of HPV will be assessed both before and 3 months after each intervention, and satisfaction will also be evaluated after the intervention in each group.

**Results:**

As of May 2025, a preliminary result enrolling 124 students showed an increase in vaccination coverage after students played a serious game. We expect higher vaccination rates in the intervention groups compared with the control group, although it is difficult to predict which strategy will be more effective. The estimated target vaccination coverage for groups b and c is 45%.

**Conclusions:**

This study aims to improve HPV vaccination rates among teenagers in Réunion Island by evaluating the impact of 2 awareness-raising strategies using innovative and engaging tools. If successful, this approach could be adapted and implemented in other regions of France or internationally.

**International Registered Report Identifier (IRRID):**

PRR1-10.2196/73366

## Introduction

Human papillomavirus (HPV) is a widespread sexually transmitted infection that affects more than 1 in 5 people globally [1]. It is estimated that about 80% of men and women will be exposed to HPV at some point in their lives. With a global prevalence of 31% in men and 32.1% in women [2,3], HPV represents a major public health concern due to its established role in the development of multiple cancers [4], most notably cervical cancer, which remains the fourth most frequent cancer in women worldwide. The World Health Organization reported 660,000 new cervical cancer cases and 350,000 related deaths in 2022. In France, HPV is responsible for over 6400 cancers annually, including cervical, vulvar, vaginal, penile, anal, and oropharyngeal cancers. On Réunion Island, cervical cancer is the third most common cancer in women.

Prevention of cervical cancer is mainly based on screening with Papanicolaou (Pap) smear tests and HPV vaccination. Despite the proven safety and effectiveness of HPV vaccines in reducing the incidence of precancerous lesions and genital warts [5-7], vaccination coverage in France remains insufficient. As of 2023, only 45% of girls and 16% of boys aged 16 were vaccinated [8]. In Réunion Island, coverage is even lower: in 2022, only 12.7% of adolescents under 16 had received at least one dose of the HPV vaccine. Following the national vaccination campaign launched in 2023, only 6.8% of seventh-grade students were vaccinated at school in Réunion. Taking into account children vaccinated outside school by their general practitioner, the rate modestly increased to 27% for girls and 16% for boys. Nevertheless, these figures remain far from the thresholds needed to achieve herd immunity and effective cancer prevention [9].

The low uptake in Réunion Island may be attributed to a lack of information, fear of adverse events, and limited engagement from both health care professionals and families [10,11]. In countries such as Australia, Belgium, Portugal, and the United Kingdom, school-based HPV vaccination programs have achieved coverage rates exceeding 80% [12]. These programs reduce access barriers, promote equity, and provide opportunities for engaging educational interventions.

Educational strategies themselves are key determinants of success. Traditional top-down health messages may be poorly received by adolescents, particularly when sensitive topics such as sexually transmitted infections are involved. In response, 2 innovative, youth-centered approaches have emerged: peer-led education (ambassador programs) and serious games.

Peer education leverages the credibility, relatability, and social dynamics of adolescence. Studies have shown that adolescents are more likely to engage with and retain information delivered by peers than by adults or authority figures. In a 2018 study conducted in Niger with more than 1000 students, peer-led HPV education significantly increased knowledge scores (from mean 12.94, SD 9.23 to mean 53.74, SD 10.69, *P*<.001) [13]. This underscores the power of peer-to-peer communication in culturally and resource-diverse settings.

In the field of health sciences, peer-assisted learning has been widely applied in clinical education. A systematic review by Markowski et al [14], analyzing 47 articles from 8 countries, concluded that peer-assisted learning improves skills, reduces stress, and increases confidence among students [14]. Importantly, these effects are not limited to the “ambassadors”—students receiving information from their peers also benefit significantly from the learning process [15].

In the context of vaccination, peer influence has been shown to enhance acceptance, particularly when combined with school-based efforts and community engagement. Peer-led interventions not only promote knowledge but also shape attitudes and behavioral intentions—factors especially relevant to HPV vaccination, which is often stigmatized or misunderstood.

Serious games—defined as games designed for a primary purpose other than pure entertainment—are increasingly used in health education. They offer immersive, interactive learning experiences that promote engagement, critical thinking, and long-term retention of knowledge. In recent years, serious games have been applied to topics ranging from antimicrobial resistance to smoking prevention.

Already in 2016, it was highlighted that serious games, through their unique combination of pedagogy and play, foster active learning, strengthen motivation, and adapt to different learner profiles. While recognizing the need for thoughtful integration into the pedagogical framework, the literature also provides tools for selecting and using these games appropriately [16].

In the French Antilles, unpublished pilot projects used serious games to address tobacco and alcohol prevention in adolescents. In mainland France, the Paris Committee and the French League Against Cancer developed a serious card game to educate students about the harms of smoking. A 2019 literature review of serious games in health education found that, while most studies focused on participant satisfaction, several also reported improvements in knowledge, decision-making, and self-efficacy [17].

A Swiss study conducted in 2020 reported behavior change following increased knowledge of COVID-19 protection measures in a small sample of employees in a long-term care unit [18].

A 2021 Irish study evaluated the impact of a serious game on influenza vaccination among nursing students. Postintervention, knowledge scores rose from 68% to 85%, and positive attitudes toward vaccination improved significantly [19]. Although data on serious games for HPV remain limited, these early findings support their potential as engaging tools for behavior change, particularly when integrated into school curricula.

Building on the findings of Tran et al [20], we hypothesize that both peer education and serious games can improve HPV vaccination uptake among adolescents. To evaluate this, we have designed a randomized controlled trial comparing 3 strategies in middle schools across Réunion Island (Textbox 1).

**Strategies in middle schools across Réunion Island.**

1. Control group

- Standard school-based vaccination campaign with no additional educational component.

2. Ambassador group

- Implementation of a peer-led education model, with trained student ambassadors delivering information to their peers.

3. Serious game group

- Delivery of a cus
  tom-designed, human papillomavirus–focused serious game to promote learning and engagement.
- As the local education authority of Réunion Island does not permit promotion of this type of intervention in virtual form, to limit screen exposure among young people, we have planned the game in a card-based format.

Comparing peer-led (ambassador) education and serious games within the same trial will allow us to determine the most effective and scalable strategy for improving HPV vaccination uptake in Réunion Island. While both approaches have shown promise in preliminary studies and international contexts, they differ in required resources, modes of engagement, and potential for long-term implementation. Peer-led education depends on sustained student involvement and social interaction, whereas serious games provide a more immersive, standardized, and potentially reproducible experience. By rigorously evaluating their respective impacts in a controlled setting, this study will inform future public health programming. Once the most effective and cost-efficient strategy is identified, efforts and resources can be directed toward its wider deployment at the regional level—and potentially beyond—to maximize public health impact and reduce HPV-related disease burden among adolescents.

By targeting students in schools—where barriers to access can be minimized—we aim to advance the long-term goal of reducing the HPV-related disease burden in underserved regions such as Réunion Island.

## Methods

### Trial Design

This is a randomized, controlled, open-label intervention study based on 2 awareness strategies for HPV vaccination in Réunion Island. A prototype of the serious game was created in May 2025, and the service company will devote several weeks to producing the final materials. The card game is expected to be ready for the start of the school year.

Colleges (selected from lists provided by the local education authority in March-April) will then be randomly assigned to 3 groups, with 8 colleges per arm:

- Reference arm: no intervention; schools will only follow the government’s vaccination campaign.
- Ambassador arm: implementation of specific HPV vaccine education to create an ambassador class.
- Serious game arm: distribution of the card-based serious game.

The unit of randomization will be the middle school. Each school will be assigned to 1 of the 3 intervention arms (1, 2, or 3) according to a randomization list. The allocation ratio will be 1:1:1, with stratification by middle school level. To minimize imbalance between schools, a constrained randomization plan will be used (PROC PLAN; SAS Institute Inc). The randomization list will be generated by a statistician at the Methodology and Data Management Center before the start of the study. A document describing the randomization procedure will be kept confidential within the same center.

The directors of the randomized schools will be invited to participate in the study. Our study is supported by the rectorship of Réunion Island, which has already written to all directors to request their assistance with this project.

Once school directors agree, they will be asked to identify 1 or 2 teachers, on a voluntary basis, to help implement the study in each school. From our experience, teachers are highly motivated to take part in public health initiatives, particularly those involving playful learning.

If directors decline participation, a new randomization will be conducted in additional schools to ensure school buy-in.

Interventions will be conducted only in seventh-grade classes, targeting children around 12 years of age.

All participants will complete an HPV baseline knowledge questionnaire before the intervention and again up to 3 months afterward.

Following the trial, the team will collect data on vaccination uptake, with initiation of the vaccination schedule (at least one dose) considered sufficient for assessment, as the World Health Organization has concluded that a single-dose HPV vaccine may provide solid protection [21].

### Study Objectives

The primary objective is to evaluate the impact of 2 awareness-raising strategies against HPV by involving middle school students to increase vaccination uptake. Specifically, we aim to compare the proportion of students who initiated vaccination before and after the interventions. In addition, we will assess motivations and challenges encountered in both the serious game and ambassador classes, as well as overall student satisfaction and knowledge.

### Settings

**Figure 1:**
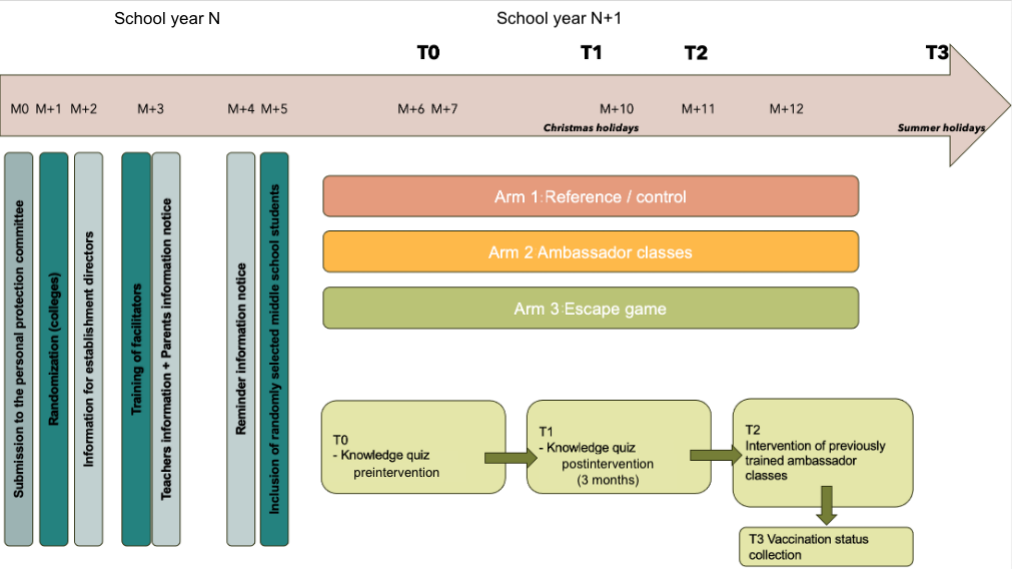
Participants timeline T0 : pre-intervention questionnaire T1 : post intervention questionnaire at 3 months T2 : only for ambassador classes : information by ambassador classes to their peers in 7th grade class T3 : collection of vaccination status.

This study is scheduled to start in August-September (the beginning of the school year) and is expected to last approximately 1 school year, concluding in July (Figure 1). The research team will perform randomization to allocate 8 colleges per arm.

In August, at the beginning of the new academic school year, parental consent will be collected.

In September, children will be enrolled after confirming that their parents do not object to participation.

Vaccination campaigns typically occur in December and June, with the first dose usually given in December and the second dose in June, although vaccination can also be initiated with the first dose in June, all scheduled before school holidays.

Between September and December, the following events will occur:

- In the ambassador group, a facilitator, after receiving prior training, will introduce HPV to students in the ambassador classes using a standardized slideshow identical across all classes. These students will then be responsible for conveying this information to other seventh-grade classes in their school. They will have the autonomy to choose the format for delivering the information to their peers (eg, games, presentations, films, posters, slideshows, comics).
- In the serious game group, 1 facilitator will explain the functioning and rules of the game to volunteer science teachers. The game is designed so that teachers can work independently with the students. Teachers may choose when to conduct the serious game during school classes before the vaccination campaigns.

At the end of the school year, around July, vaccination status will be collected using the French social security system, following anonymization of social security numbers.

### Ethics Approval and Consent to Participate Statement

The participants of this study are minors. This intervention study does not involve administering the HPV vaccine but focuses on raising awareness. The research team will inform parents about the study via an information leaflet distributed in June and August, and written consent will be obtained unless parents object.

The sponsor and the investigator(s) commit to conducting this research in compliance with French law number 2012-300 of March 5, 2012, relating to research involving human participants, as well as in accordance with Good Clinical Practice (International Council for Harmonisation, version 4, November 9, 2016; and decision of November 24, 2006) and the Declaration of Helsinki (full text available online [22]).

The research will be conducted in accordance with the present protocol. Except in emergency situations requiring the implementation of specific therapeutic procedures, the investigator(s) commit(s) to fully comply with the protocol, particularly with respect to obtaining consent and the reporting and follow-up of serious adverse events.

Data collected during this research will be processed by the Centre Hospitalier Universitaire de La Réunion in compliance with the French Data Protection Act no. 78-17 of January 6, 1978, as amended by Act 2004-801 of August 6, 2004.

Approval from the ethics committee and the data protection authority is currently in progress.

### Patient and Public Involvement

Following the distribution of information leaflets to parents and students, and after obtaining consent from school directors, the schools will be randomized. The research team, together with teachers, will verify any potential objections and ensure that students whose parents object do not participate in the interventions. Parents may withdraw their child from the study at any time by simple request. Participants will have the right to receive information about the study, including the results.

### Sample Size

This is a cluster randomized controlled trial with 3 parallel arms, designed to evaluate the effectiveness of 2 awareness programs addressing HPV-related risks and its prevention in seventh-grade classes in Réunion colleges. The reference arm is a no-intervention group. The allocation ratio is 1:1:1. The primary outcome is the proportion of students who initiate the HPV vaccination schedule. The World Health Organization Strategic Advisory Group of Experts has recently reviewed evidence indicating that a single-dose vaccination schedule provides effectiveness comparable to the traditional 2-dose schedule.

**Figure 2:**
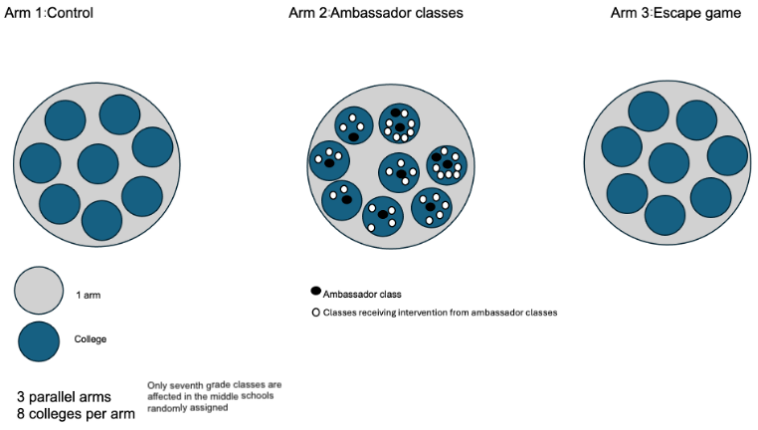
Diagram showing the composition of colleges randomly assigned.

The research team may include up to 1 additional ambassador class in arm 2 if a college has more than 5 seventh-grade classes, to facilitate information dissemination (Figure 2).

To calculate the required number of participants, the following assumptions are considered:

- It is hypothesized that, by the end of the school year, 30% of students in the control group will have initiated vaccination. This estimate is based on vaccination coverage data published by Santé Publique France in 2023, which reported that 21.5% of children (girls and boys combined) aged 12 in Réunion had received at least one dose of the HPV vaccine by December 31, 2023 [23].
- It is hypothesized that the ambassador classes and the serious game will increase coverage to 45% in the respective intervention groups.
- A constant intracluster correlation coefficient of 0.1, considered a rather conservative value [24].
- An α risk of 5%
- 90% power
- The average number of seventh-grade students (n) is 190 per college.
- A design effect (allowing intraclass correlation [ICC] to be taken into account) of 19.9, which is calculated as 1 + ICC × (n – 1).

Therefore, a total of 8 colleges per arm will be included, resulting in 24 colleges overall and approximately 3144 students.

### Recruitment

Our sample will include seventh-grade middle school students from randomly selected schools in Réunion Island, following approval from the school directors. These schools will be randomly assigned to 1 of the 3 groups in a 1:1:1 ratio using a randomization algorithm. All students attending the selected schools will be invited to participate in the study (Figure 2).

### Eligibility Criteria

Inclusion criteria are students in seventh-grade classes from middle schools in Réunion Island who are randomly selected and assigned to the study.

Exclusion criteria are students with parental opposition to participating.

### Intervention

#### Control Group

The control group will consist of 8 randomly assigned middle schools. These schools will follow the existing vaccination campaign, without any modifications, as implemented by the French government. As vaccination campaigns are coordinated by the local Regional Health Agency, they are relatively uniform across all middle schools in Réunion, ensuring standardization and internal validity. Parents will receive information materials and consent forms through their children. Both parents are invited to sign the written consent form, which the child will then submit to the school counselor or school nurse. The counselor or nurse will forward the forms to the vaccination teams assigned to the area. This allows the vaccination teams, in coordination with the education staff, to schedule their visit to the school on a specific day and bring the appropriate number of vaccine doses corresponding to the consent forms received. Vaccination teams typically visit each school twice a year to administer 2 vaccine doses 6 months apart, usually in December (year Y) and June (year Y+1).

A preintervention questionnaire will be administered to assess students’ baseline knowledge about HPV (T0). The questionnaire will be standardized, anonymous, and identical for all participants. It will be available on tablets, computers, or on paper, depending on availability. A postintervention knowledge assessment will be conducted 3 months later (T1) using a questionnaire identical to the preintervention one, to evaluate changes in the rate of correct responses before and after the vaccination campaign.

The standardized knowledge questionnaire was developed by the research team and will assess students’ knowledge of HPV epidemiology, complications, and prevention (including vaccination; Multimedia Appendix 1). Each question requires a response of “yes,” “no,” or “I don’t know.” The questionnaire consists of 11 questions.

Vaccination status will be collected using the French social security system after anonymization of social security numbers (T3).

#### Intervention Group: Ambassador Class (Peer Learning)

For each college randomized to this arm, 1 seventh-grade class will be randomly selected as the ambassador class. If a college has more than 5 seventh-grade classes, 2 ambassador classes will be designated (Figure 2). Students in the ambassador class will receive the intervention during a single class period.

This group will receive a 30-minute presentation on HPV delivered by a trained facilitator during class. The presentation will be standardized and consistent across all participating classes to minimize potential bias. Developed in advance by a team of experts, it will remain identical for all classes in this intervention arm. The presentation will cover key topics, including HPV, its epidemiology in France and Réunion Island, its benign and malignant sequelae, and preventive measures such as screening and vaccination. To assess students’ understanding of the material, they will complete a satisfaction questionnaire following the presentation.

The same preintervention (T0) and postintervention (T1) questionnaires used in the control group to assess HPV knowledge will be administered.

This arm, which integrates peer learning, will train “ambassador classes” on HPV prevention. In a second step, these students will disseminate the information to their peers in other seventh-grade classes within their middle school (T2). They will have the autonomy to choose the format for delivering the information during class (eg, games, presentations, films, posters, slideshows, comics [20]). Students who receive the information from their peers will also complete a satisfaction questionnaire. A field coordinator (a volunteer teacher) will be present during the training sessions to ensure that essential information is effectively conveyed and, if necessary, to supplement any important details that may have been omitted in the student-led presentations.

Vaccination status will be collected using the French social security system after anonymization of social security numbers (T3).

#### Intervention Group: Serious Game Cards

The intervention for this group will involve a card game modeled after an “unlock” serious game, specifically designed for this study to raise awareness about HPV. All seventh-grade classes in the randomized middle schools will participate in the game during a 1-hour class session. Sufficient sets of the card game will be provided to ensure that each student can actively participate. Middle school students will be supervised by a volunteer science teacher.

The game was designed to be played directly by students working in teams.

The game consists of 3 types of cards:

- Yellow cards: feature fictional child characters expressing their own doubts about the HPV vaccine, as well as concerns raised by their parents.
- Blue cards: provide answers addressing the children’s doubts or fears.
- Green cards: respond specifically to the concerns expressed by parents.

**Figure 3:**
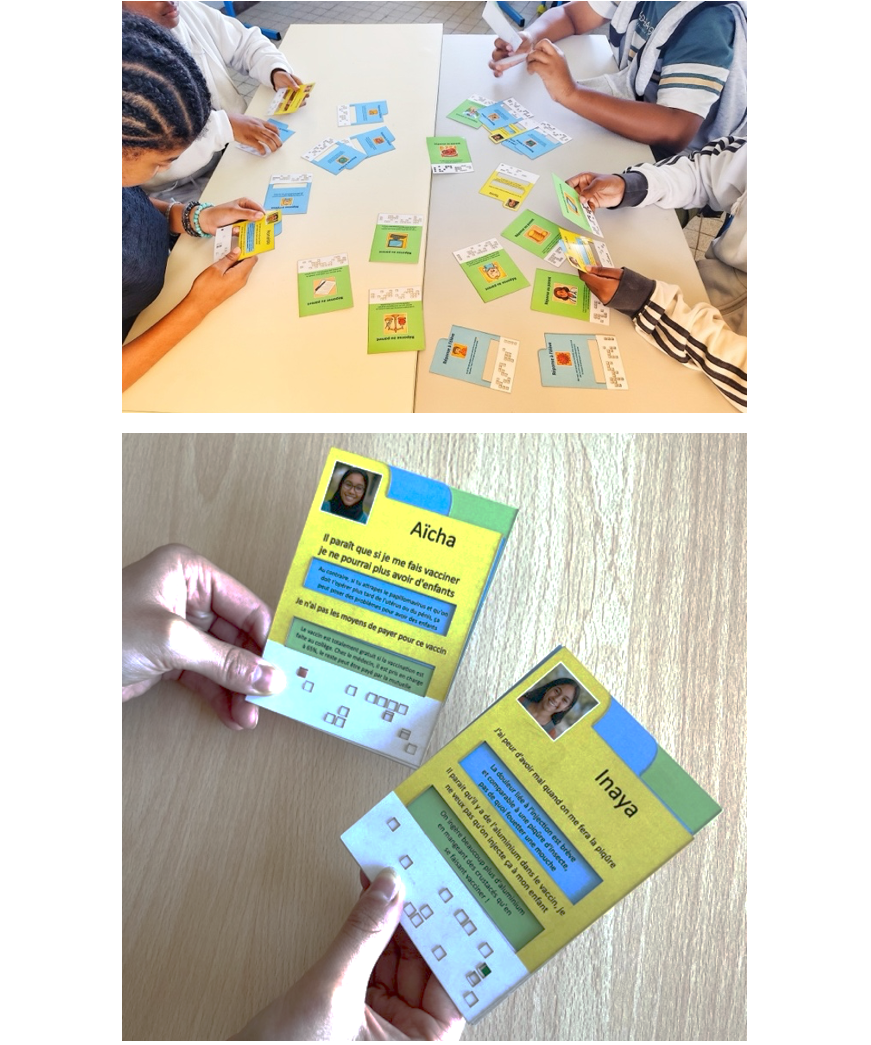
Serious game about HPV vaccination.

First, the teacher reads the game instructions and mission order to the students for 5 minutes. A deck of cards is then distributed to groups of 3-4 students. According to the game rules, students must form a correct trio by matching 1 yellow card with 1 blue card and 1 green card (Figure 3). Students have 45 minutes to solve as many card trios as possible by finding the correct matches. Each correct answer represents 1 vaccinated child in their class and must be validated by the teacher. The final objective is to “vaccinate” as many children as possible.

The intervention is grounded in a participatory communication approach that fosters active engagement among students. Children lead the game themselves, encouraging peer interaction and collaborative learning.

Children and parents were not involved in designing this game. However, the doubts featured on the yellow cards were derived from a study conducted a few years ago to identify barriers to HPV vaccination in schools [11]. A pilot evaluation of the game was carried out in 1 middle school, which helped identify areas for improvement and ensured smoother implementation.

At the end of the allotted time, teachers and students can debrief together to reinforce key concepts about HPV, and the teacher can answer any questions. The remaining 5 minutes of class time are allocated for students to complete the satisfaction questionnaire.

The same preintervention (T0) and postintervention (T1) questionnaires used in the control group to assess HPV knowledge will be administered.

In addition, students will complete a satisfaction questionnaire following the game, which will take a maximum of 5 minutes.

Vaccination status will be collected using the French social security system after anonymization of social security numbers (T3).

### Measures

#### Primary Outcome: Evaluate New Tools to Improve Vaccination

The primary outcome of this study is the proportion of students in each group who initiate HPV vaccination by the end of the school year (at least one dose of the HPV vaccine).

#### Secondary Outcomes

The secondary outcomes are as follows:

- Evaluate the proportion of girls and boys in each group who initiate HPV vaccination by the end of the school year.
- Explore and assess the motivations and challenges perceived by students in the ambassador classes and by those participating in the serious games.
- Evaluate overall student satisfaction within each arm for descriptive purposes.
- Compare students’ knowledge before and after the intervention in each arm.

#### Blinding

Given the nature of the interventions, blinding will not be implemented in this study. No participants will be blinded during the trial.

### Data Collection, Management, and Monitoring

Pre- and postintervention knowledge questionnaires will be identical for all students (Multimedia Appendix 1). Satisfaction questionnaires will be tailored to the specific trial arm—ambassador class or serious game—to evaluate the unique aspects of each intervention (Multimedia Appendices 2 and 3). Data collection via questionnaires will be anonymized and conducted at the class level.

Vaccination status will be collected with the assistance of the French social security system, following anonymization of social security numbers.

All information required by the protocol must be recorded in the observation notebooks, and any missing data must be explained. Data should be collected as they are obtained and transcribed neatly and legibly. Data will be entered into an electronic observation notebook (electronic case report form) using Ennov Clinical software.

A clinical research associate appointed by the sponsor will regularly visit the investigator center—at study initiation, periodically during the research depending on the inclusion rate, and at study completion. During these visits, and in accordance with the risk-based monitoring plan (considering participants, logistics, impact, and resources), the following elements will be reviewed:

- Nonopposition from participants
- Compliance with the research protocol and defined procedures
- Quality of data collected in the observation notebook, including accuracy, completeness, and consistency with source documents (eg, medical records, appointment books, original laboratory results)

A written monitoring report will be prepared for each visit.

An audit may be conducted at any time by individuals mandated by the sponsor and independent of the research team. Its purpose is to verify participant safety and rights, ensure compliance with applicable regulations, and assess the reliability of the data.

An inspection may also be ordered by a competent authority (eg, ANSM [Agence nationale de sécurité du médicament et des produits de santé] in France).

Both audits and inspections may be conducted at any stage of the research, from protocol development to publication of results and the handling of data generated during the study.

Investigators agree to comply with the sponsor’s requirements for audits and with the competent authority’s requirements for inspections.

Four follow-up visits will be scheduled (Textbox 2).

**Follow-up visits.**

1. Visit 1 (T0)

- This visit will take place during school hours, just before the start of the awareness program (arms 2 and 3).
- Students will complete a questionnaire assessing their knowledge of human papillomavirus (HPV).
- The estimated completion time is a maximum of 10 minutes.

2. Visit 2 (T1)

- This visit will take place during school hours, approximately 3 months after the end of the awareness program.
- Students will complete the same questionnaire to assess their postintervention knowledge.
- The estimated completion time is a maximum of 10 minutes.

3. Visit 3 (T2)

- This visit will take place only in the middle schools assigned to the “ambassador classes” arm to assess the satisfaction of students receiving the intervention from their trained peers.
- A member of the research team will be present during the ambassador class intervention to ensure that essential information is accurately conveyed.
- The estimated completion time is a maximum of 10 minutes.

4. Visit 4 (T3)

- At the end of the study, qualitative interviews will be conducted to explore the motivations and challenges experienced by teachers, parents, and students participating in the study.
- Each participant will receive information about the study and will provide consent for audio-recording of their interview.
- The interviews will then be transcribed anonymously using qualitative data analysis software.

5. End of research visit

- The end-of-research visit marks the final data collection, which may include qualitative interviews or vaccination data.
- This visit will also be used to collect information on HPV vaccination initiation during the school year (at least one dose counts) for each student, with assistance from the Social Security system via anonymized social security numbers.
- Within 1 year of the end of the research or its early termination, a final report will be prepared and signed by both the sponsor and the investigator.
- This report will be submitted to the competent authority.
- Additionally, the sponsor will provide the Committee for the Protection of Persons with a summary of the final report within 1 year of the study’s conclusion.

### Statistical Analysis

Analyses will be performed by the biostatistics team at La Réunion University Hospital, in collaboration with the study methodologist and coordinating investigator, according to the statistical analysis plan established before database lock. Analyses will be conducted using SAS (version 9.4; SAS Institute Inc), Stata (version 16; StataCorp), or R (R Foundation) software.

Qualitative variables will be described using counts and percentages. Quantitative variables will be summarized using the mean and SD, or the median and range, as appropriate.

Comparative analyses of the end points will be conducted according to the intention-to-treat principle, meaning that students will be analyzed in the group to which their class was randomized, regardless of any deviations from the research protocol. All students included and followed until the end of the school year will be considered in the analysis.

At the college level, the main deviation from the expected research protocol is nonparticipation in the intervention allocated by randomization.

At the student level, expected deviations from the protocol include occasional absenteeism during data collection at different periods, permanent departure from college during the academic year, or individual refusal to participate at any point during the study. These factors may lead to a loss of statistical power and could affect the generalizability of the findings. Statistical significance will be set at *P*<.05 (2-sided).

The results of this study will be published in accordance with the CONSORT (Consolidated Standards of Reporting Trials) recommendations extended to cluster randomized trials.

## Results

### Preliminary Findings

A first draft of the serious game was created with funding obtained in March 2024 (Innovation Prize of University Hospitals).

As of May 2025, it was tested in 1 middle school in Réunion Island across all seventh grades, with the approval of the school director and teachers, before the vaccination campaign. The game is intended to be used by teachers alone, without the help of a game animator, to enhance reproducibility.

Children were engaged by the game, and their intention to vaccinate was higher than in other schools. Among the 189 students in seventh grade at that school, vaccination teams had initially received consent for 10 students to be vaccinated. After the intervention/serious game, more students submitted signed consents, and a total of 25 (13.2%) students were vaccinated. In other schools in Réunion during that campaign, vaccination coverage did not exceed 7% (986/14,500, 6.8%).

Although the serious game has been developed, some minor changes need to be considered following this first experience with children. Once finalized, production of several copies will be launched. As registrations are in progress, the study is expected to start during the academic year in August 2026.

### Expected Outcomes

Réunion Island has one of the lowest vaccination coverage rates among regions in France and Europe (14.8% overall, with 16.1% among girls and 3.4% among boys). Within 1 year, we expect this coverage to remain stable or only slightly increase. We hypothesize that by the end of the school year, 30% of children in the control group will have initiated vaccination—based on data published by Santé Publique France in 2023, which reported that 21.5% of 12-year-old children (girls and boys combined) in Réunion had received at least one dose of the HPV vaccine by December 31, 2023.

With this study protocol, we aim to achieve a vaccination coverage rate of around 45% among children through the proposed interventions (ambassador classes and serious games). This target was used to calculate the sample size. It is not yet known which of the 2 strategies will be more effective.

This study protocol could be implemented in other departments. By validating innovative strategies to improve HPV vaccination coverage in middle schools, this research could not only influence national vaccination campaigns but also provide insights for similar initiatives in other medical fields. It could play a central role in combating vaccine hesitancy, particularly among younger generations, by informing the population. The study results may also positively impact the involvement of children and families in vaccination campaigns. By reinforcing messages delivered by health care professionals and improving communication with families of participating children, this study could support greater acceptance of the HPV vaccine, thereby reducing the incidence of benign, precancerous, and cancerous conditions associated with the virus.

## Discussion

### Comparison With Previous Work

As mentioned earlier in the introduction, high levels of vaccination coverage are achieved in countries that vaccinate in schools [12]. In 2020, a team in La Réunion increased vaccination rates following interventions, with 26 girls completing the vaccination schedule compared with 3 boys [25].

Peer learning involves students acquiring knowledge or skills through experience, observation, or education [15]. This method allows students to think collectively about a subject, enhancing their critical thinking and understanding. Studies have shown potential benefits of this learning process in clinical training for adults, as well as positive social and academic gains among children. It reduces stress, increases self-confidence, and improves skills [14,26], not only in ambassador students but also among their peers who benefit from the ambassadors’ knowledge [13].

Serious games have been developed as educational tools in recent years. They are immersive games built around a scenario and typically involve solving puzzles within a predetermined time frame. In the health field, serious games were introduced in 2017 to improve knowledge, increase interest and motivation, and enhance team cohesion and communication. However, literature on the subject is scarce, and although participant perception has been evaluated, data on knowledge improvement were not provided [17].

### Study Limitations

Vaccination coverage is expected to continue increasing between 2023 and 2025, which may influence the number of schools included in each study arm and affect the calculation of the required sample size. This trend could potentially reduce the study’s statistical power.

The potential for cross-talk between students in the intervention and control groups may introduce bias in this open/unblinded study and lead to contamination between students, especially in a setting like Réunion Island, where communities can be tightly knit and students may interact across schools or through extracurricular activities.

Interventions cannot be staggered over time because they depend on the academic calendar, which is punctuated by school holidays.

Nevertheless, to minimize contamination between the groups:

- We chose to use schools as units of randomization, rather than individual students.
- We will try to select schools that are geographically separate—ideally from different cities—to limit student interaction, although this remains subject to randomization.
- We will try to measure the extent of contamination at the end of the study by asking students whether they heard about HPV vaccination from students in other schools.
- Although blinding is not practically feasible for this study, students and teachers in control schools can be informed that they are part of a general public health campaign without knowing the specific educational strategies tested in other arms. We have planned an objective primary end point to compensate for this. Recovery of vaccination status will be carried out using the French social security system after anonymization of social security numbers.
- We plan continuous monitoring in the form of visits to establish quality controls throughout the study, including on-site visits and calls to school officials.
- We will use a mixed logistic model with random intercepts to assess the effect of the 2 interventions, compared with the control group, on the proportion of vaccinated individuals. This model will allow us to account for the cluster structure of the data. By including schools as random effects, we consider that each school may have a different baseline proportion of vaccinated individuals due to unmeasured factors. The interventions will be modeled as fixed effects. A mixed logistic model with a random intercept is an ideal approach for analyzing data in a cluster-randomized study (such as one involving schools) while accounting for contamination bias.

### Study Strengths

To the best of our knowledge, this study is the first of its kind in France, enhancing the program recently launched by the French government for the 2023 school year by incorporating innovative educational tools, such as serious games, in comparison with peer learning through ambassador classes. The results of this study, along with the development of these educational programs, could be adapted and applied to other regions in France—or even to other countries—for different medical subjects.

In addition, the sample size allows us to achieve a higher level of confidence.

This study focuses on a major public health issue in the current epidemiological context. It is important to address this now, given the effectiveness of the vaccine in preventing papillomavirus-related complications. The study will help identify the life stage during which vaccination is most effective and guide targeted primary prevention efforts.

If this study demonstrates effectiveness, the expected impact will be significant, providing numerous benefits for public health in the short, medium, and long term:

- Validate the most effective strategies for improving HPV vaccination campaigns in French schools and potentially internationally.
- Increase HPV vaccination coverage in Réunion Island.
- Optimize school-based vaccination campaigns.
- Promote the involvement of students in the HPV vaccination campaign across all Réunion colleges.
- Enhance and reinforce the messages delivered by health care professionals and public communication channels.
- Raise awareness among the families of participating children.
- Reduce the incidence of HPV-related benign conditions, such as condyloma, as well as precancerous and cancerous lesions.

### Conclusion

To the best of our knowledge, this represents the first study aimed at enhancing HPV vaccination rates among children in Réunion Island using innovative tools, specifically serious games and ambassador-led classes. This multicenter study may offer promising intervention strategies to address HPV vaccine hesitancy and could potentially complement the ongoing HPV vaccination campaign in France. By rigorously evaluating their respective impacts within a controlled setting, this study will inform future public health programming. Once the most effective and cost-efficient strategy is identified, efforts and resources can be focused on its broader implementation at the regional level—and potentially beyond—to maximize public health benefits and reduce the burden of HPV-related disease among adolescents.

## Appendix

Multimedia Appendix 1 Knowledge quiz.

Multimedia Appendix 2 Satisfaction questionnaire for ambassador classes.

Multimedia Appendix 3 Satisfaction questionnaire for the escape game group.

## Abbreviations

ANSM: Agence nationale de sécurité du médicament et des produits de santé
CONSORT: Consolidated Standards of Reporting Trials
HPV: human papillomavirus
ICC: intraclass correlation
Pap: Papanicolaou
