# Supplementary material for: Boosting Human Papillomavirus Vaccination Rates: Protocol for a Randomized Controlled Trial of Awareness Interventions in Réunion Island
Source: JMIR Res Protoc. 2025 Oct 27;14:e73366. doi: 10.2196/73366 (PMC12603582; doi:10.2196/73366)
Supplement: Multimedia Appendix 1 [file resprot_v14i1e73366_app1.docx]

***Appendix 1:*** Knowledge quiz (for all arms, to be distributed at the beginning of school year and 3 months after)

***
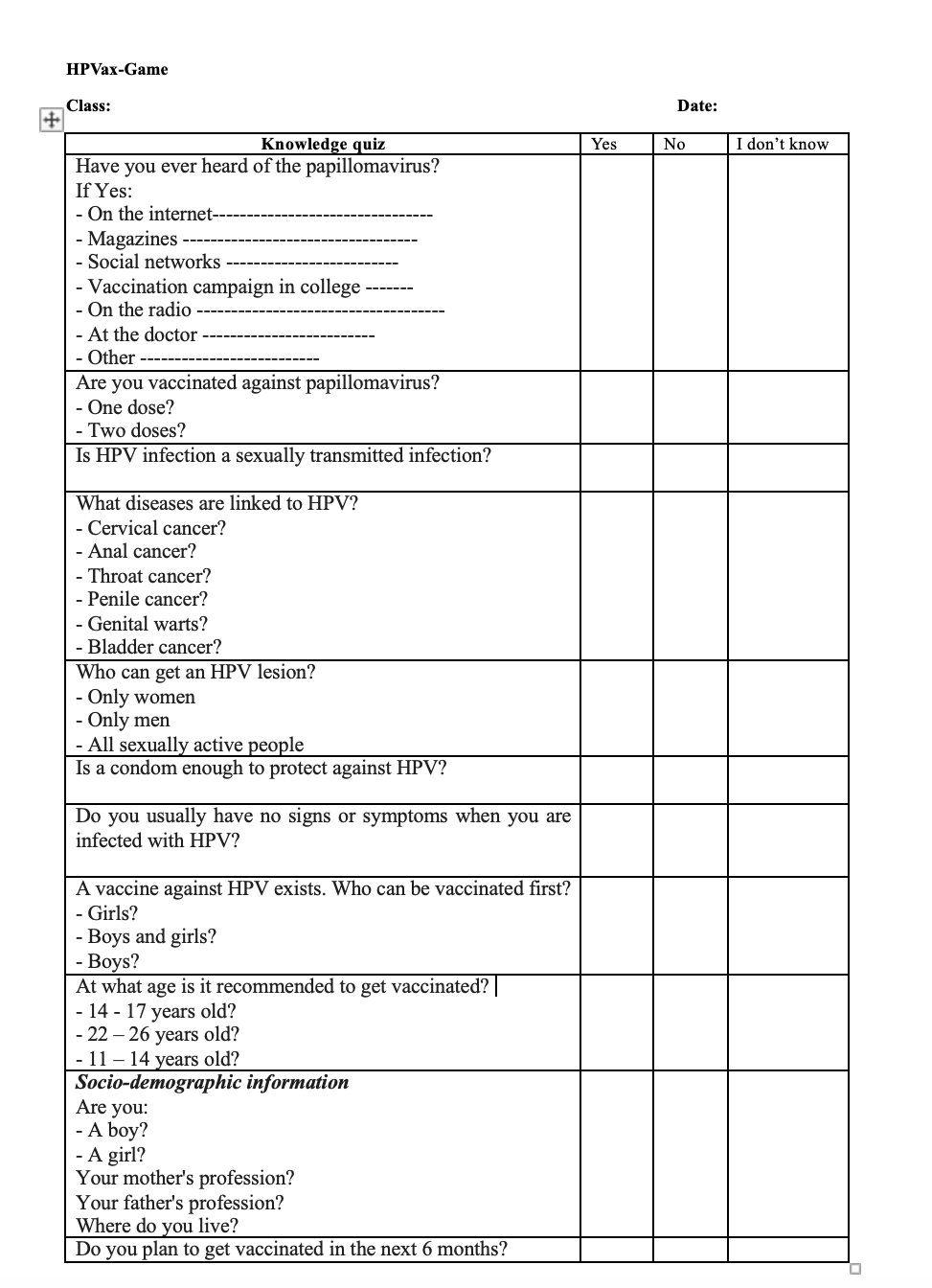
***
