# Supplementary figures and images for: Boosting Human Papillomavirus Vaccination Rates: Protocol for a Randomized Controlled Trial of Awareness Interventions in Réunion Island
Source: JMIR Res Protoc. 2025 Oct 27;14:e73366. doi: 10.2196/73366 (PMC12603582; doi:10.2196/73366)

***Appendix 2.*** Satisfaction questionnaire for ambassador classes

***
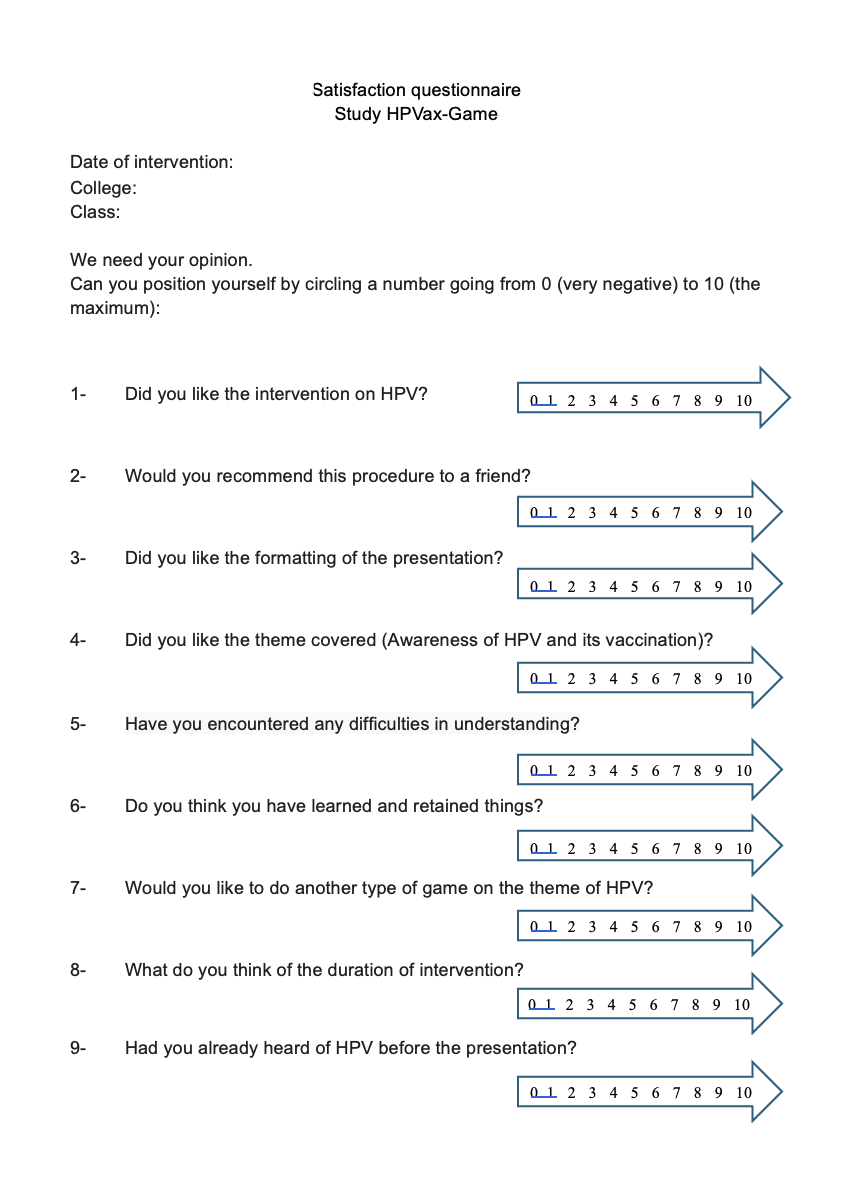
***

Supplement: Multimedia Appendix 2 [file resprot_v14i1e73366_app2.docx]

***Appendix 3.*** Satisfaction questionnaire for escape game group

***
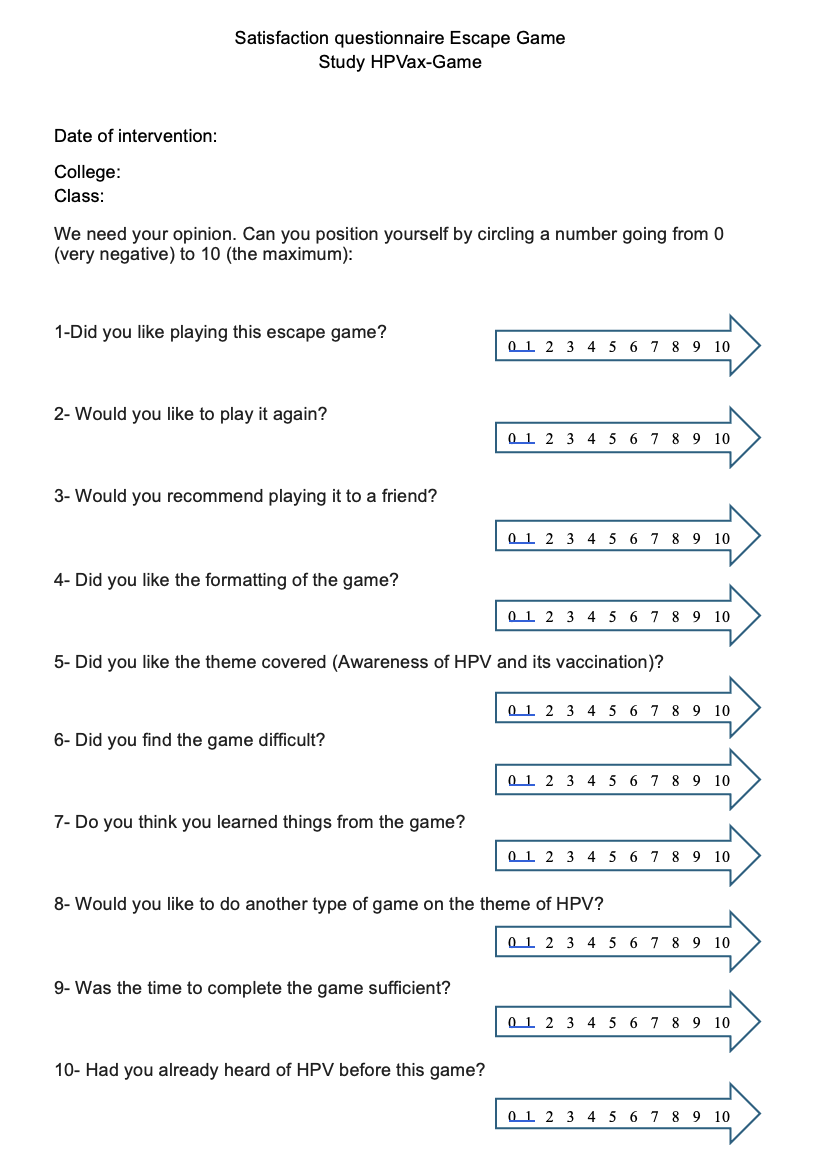
***

Supplement: Multimedia Appendix 3 [file resprot_v14i1e73366_app3.docx]
